# From Target-Oriented to Motif-Oriented: A Case Study on Nannocystin Total Synthesis

**DOI:** 10.3390/molecules25225327

**Published:** 2020-11-15

**Authors:** Weicheng Zhang

**Affiliations:** The State Key Laboratory of Medicinal Chemical Biology, College of Pharmacy, and Tianjin Key Laboratory of Molecular Drug Research, Nankai University, Tianjin 300353, China; zhangweicheng@nankai.edu.cn

**Keywords:** total synthesis, motif-oriented, nannocystin, macrocyclization

## Abstract

Natural product total synthesis is in essence target-oriented in that a set of organic transformations are orchestrated into a workable process, leading ultimately to the target molecule with a predefined architecture. For a bioactive lead, proof of synthetic viability is merely the beginning. Ensuing effort repurposes the initial synthesis for structural diversification in order to probe structure-activity relationship (SAR). Yet accessibility is not equal to flexibility; moving from convergency to divergency, it is not always feasible to explore the chemical space around a particular substructure of interest simply by tweaking an established route. In this situation, the motif-oriented strategy becomes a superior choice, which gives priority to synthetic flexibility at the concerned site such that a route is adopted only if it is capable of implementing diversification therein. This strategy was recently devised by Fürstner et al., enabling them to achieve total synthesis of both natural and non-natural nannocystins varied at an otherwise challenging position. The present review examines seven distinctive nannocystin total syntheses reported thus far and showcases the merits of conventional (target-oriented) as well as motif-oriented strategies, concluding that these two approaches complement each other and are both indispensable for natural product based drug discovery.

## 1. Introduction

**Figure 1:**
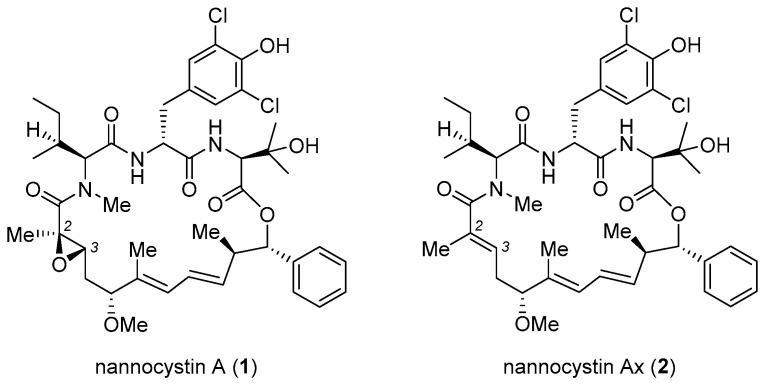
Structures of nannocystin A (**1**) and nannocystin Ax (**2**).

**Figure 2:**
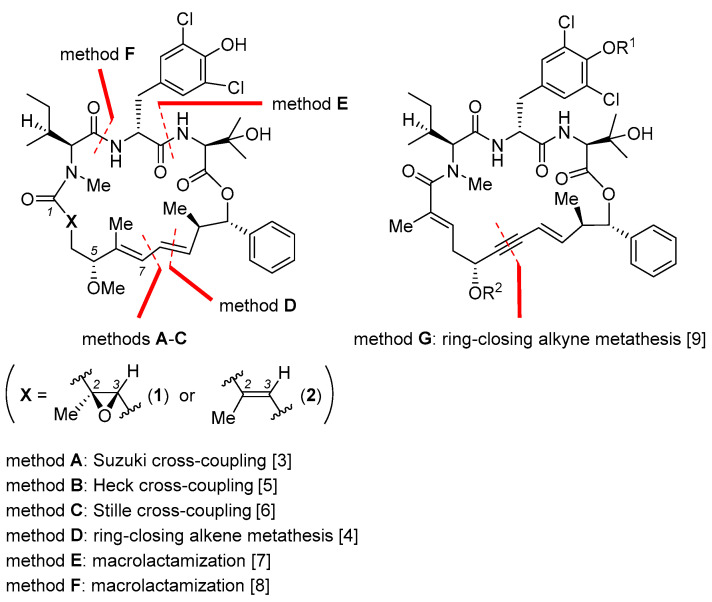
Key macrocyclization reactions (methods **A**–**G**) employed for the total synthesis of **1** and/or **2** [3,4,5,6,7,8,9].

Nannocystin A (**1**, Figure 1) and its natural congener nannocystin Ax (**2**) are myxobacterial secondary metabolites with excellent anticancer potency, which target eukaryotic elongation factor 1A (eEF1A) [1,2]. Structurally, **1** features a rigid 21-membered macrocycle adorned with nine chiral centers, two conjugated *E*-alkenes, as well as an *N*-methyl α,β-epoxy amide. Its strong antiproliferative activity, novel macrocyclic architecture, and distinct mode of action spurred endeavors from several labs to explore total synthesis. Apart from addressing the supply issue, synthetic access to **1** empowers more freedom of structural modification than biotechnological (fermentation) process. On behalf of drug discovery, it is not just a prerequisite for SAR study, but a vital way to fine-tune the many physicochemical properties of a natural lead during preclinical development. Thus far seven total syntheses of **1** or **2** have been accomplished [3,4,5,6,7,8,9], with a short summary appearing recently [10]. For efficient macrocycle generation, all synthetic routes embody the principle of convergency. Namely, synchronous preparation of a few discrete fragments precedes their sequential incorporation into a linear precursor suited for a final macrocyclization. Making a good case for the versatility and flexibility of organic synthesis, each approach deploys a unique reaction type and/or ring closure site to forge the 21-membered ring (methods **A**–**G**, Figure 2), as will be detailed below. 

## 2. Macrocyclization via Suzuki Cross-Coupling

**Scheme 1:**
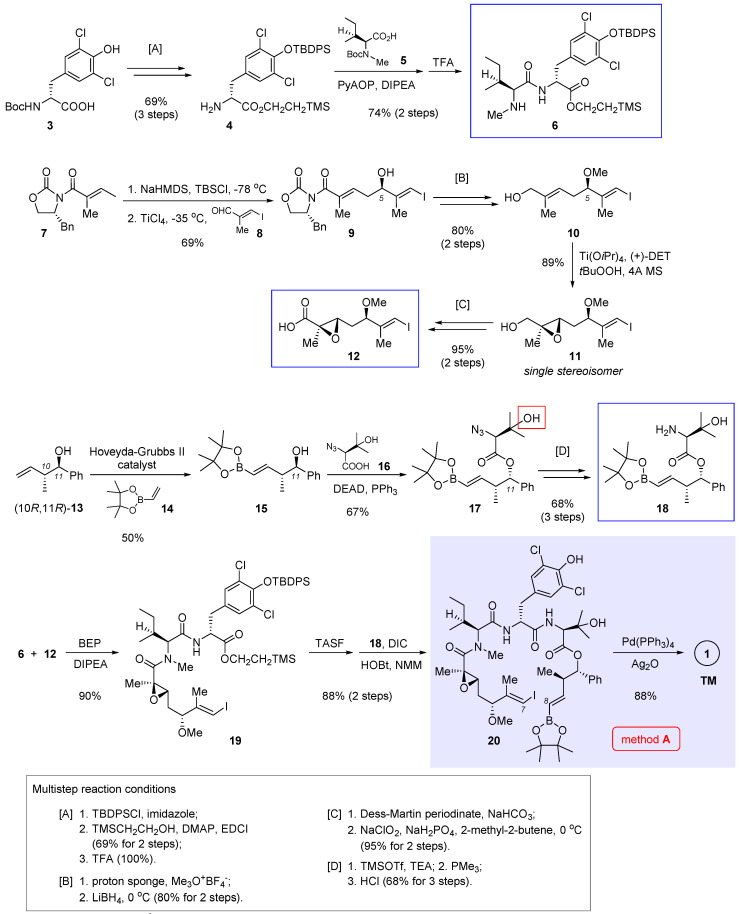
Total synthesis of **1** by Xu and Ye et al. featuring macrocyclization via Suzuki cross-coupling (Figure 2, method **A**) [3].

In 2016, Xu and Ye et al. reported total synthesis of **1** featuring macrocyclization via Suzuki cross-coupling (Figure 2, method **A**) [3]. In their retrosynthetic analysis, the final ring closure was planned to join the C7 and C8 termini of **20** (nannocystin numbering) in a stereodefined fashion. To access the open-chain precursor **20**, it is necessary to prepare three key building blocks **6**, **12**, and **18** (boxed in Scheme 1) beforehand.

Starting with commercially available L-tyrosine derivative **3**, a three-step protecting group manipulation (multistep reaction condition [A] at the bottom of Scheme 1) revealed the free amine of **4** for subsequent amide coupling with *N*-Boc-*N*-methyl-L-isoleucine **5**. Boc deprotection gave dipeptide **6** as the first building block. Next, TiCl_4_-mediated vinylogous Mukaiyama aldol condensation [11,12,13,14] was implemented between a vinylketene silyl *N*,*O*-acetal derived from **7** and (*E*)-3-iodo-2-methylacrylaldehyde (**8**), affording chiral secondary alcohol **9** in 69% yield. Methylation of the nascent 5*R* hydroxyl group, followed by reductive cleavage of the oxazolidinone auxiliary ([B]), gave rise to allylic alcohol **10**. Then Sharpless asymmetric epoxidation came into play and furnished chiral epoxide **11** as a single stereoisomer. Such prepared chiral epoxy alcohol was further converted to the second key fragment **12** via stepwise oxidation ([C]). 

Preparation of the third building block **18** was initiated by olefin cross-metathesis of the known homoallylic alcohol (10*R*,11*R*)-**13** and pinacol boronate **14** with the help of Hoveyda-Grubbs II catalyst. The resulting **15** was united with **16** via Mitsunobu esterification whereby the C11 chirality was inverted. In the next step, it seems surefire at first sight to obtain **18** directly through Staudinger reduction of the azide group present in **17**; rather, the authors isolated an unwanted epoxide as the predominant product, presumably caused by the neighboring β-hydroxyl group (highlighted in **17**). Therefore, this interfering group was masked ad hoc as a TMS ether prior to azide reduction. Acid hydrolysis of the TMS group led to **18** thereafter ([D]).

With all three essential pieces in hand, they were linked up via two amidation reactions. The first coupling between **6** and **12** is nontrivial, as a variety of common condensation reagents such as EDCI, HATU and PyAOP failed to create the desired amide bond. Fortunately, 2-bromo-1-ethyl pyridinium tetrafluoroborate (BEP) turned out superior, bringing about the coupling product **19** in a satisfactory yield (90%). Unmasking the carboxylic acid of **19** with TASF allowed the second amidation to deliver the penultimate intermediate **20**. Finally, in the presence of Pd(PPh_3_)_4_ and Ag_2_O, an intramolecular Suzuki cross-coupling closed the macrocycle, thereby attaining total synthesis of nannocystin A (Scheme 1).

## 3. Macrocyclization via Heck Cross-Coupling

Parallel to Xu and Ye’s work, we independently achieved total synthesis of **1** [5]. Central to our approach is an intramolecular Heck cross-coupling to forge the strategic C7-C8 bond (Figure 2, method **B**). Owing to unfavorable entropy loss and competition from intermolecular reactions, macrocyclization is oftentimes among the most demanding steps in macrocycle synthesis. The rationale behind our retrosynthetic deconstruction is to exploit a less sterically congestive site. In this way, we anticipated the enthalpy barrier to overcome could be lowered during the macrocycle-forming process. According to the crystal structures of nannocystin A (**1**) [2] and its phenolic methyl ether [1], we felt the polyketide C7-C8 bond stitching two *trans* alkenes might be amenable to a Heck-type cyclization. Compared with alternative Pd-catalyzed cross-coupling reactions such as the Suzuki cross-coupling utilized in the preceding synthesis (method **A**, Scheme 1) [3], Heck reaction saves the need to preassemble a latent olefinic boronate handle, thus shortening the whole route. However, a patent concern implicated by this plan lies in the uncertainty of its stereochemical outcome. In other words, unless put into practice, it is difficult, if not impossible, to tell a priori whether the cyclized product would bear the desired 8*E*-alkene, or exist as a mixed 8*Z*- and 8*E-*stereoisomers, or even worse, show up exclusively with the opposite 8*Z* configuration.

**Scheme 2:**
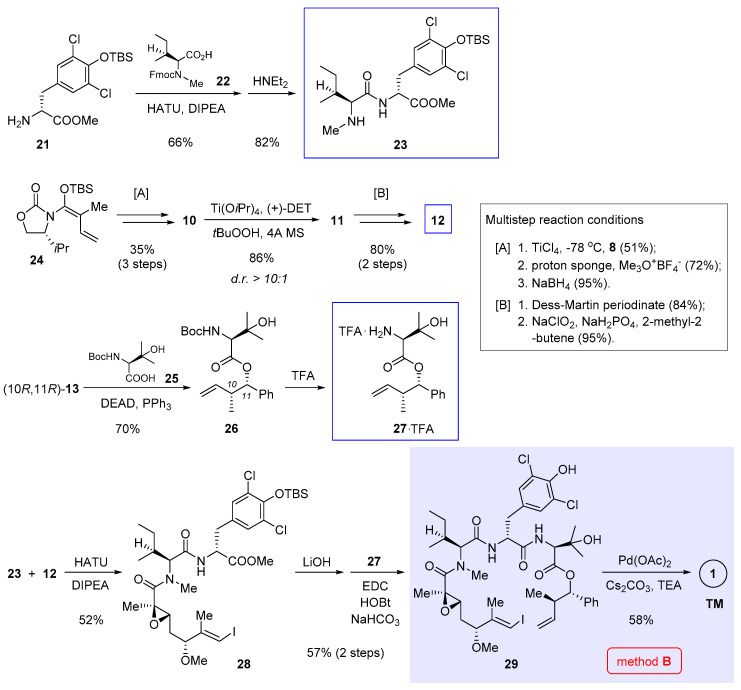
Total synthesis of **1** by Zhang and Chen et al. featuring macrocyclization via Heck cross-coupling (Figure 2, method **B**) [5].

Determined to explore the feasibility of this idea, we set out (Scheme 2) by preparing dipeptide **23** via amidation of tyrosine derivative **21** with **22** and subsequent cleavage of the Fmoc group. Meanwhile, chiral α,β-epoxy carboxylic acid **12** (structure shown in Scheme 1) was synthesized in 6 steps, involving vinylogous Mukaiyama aldol condensation [11] and Sharpless asymmetric epoxidation to create three stereocenters (multistep reaction conditions [A] and [B] compiled at the right of Scheme 2). Entry to **27** was secured through Mitsunobu esterification of (10*R*,11*R*)-**13** (structure in Scheme 1) with **25** followed by Boc deprotection. We also tried conventional ester condensation of the diastereomeric (10*R*,11*S*)-**13** and **25**, but the reaction was too sluggish to yield **26**. 

Having prepared all three necessary building blocks **23**, **12**, and **27**, we advanced to the linear intermediate **29** by first joining **23** and **12** via amidation, and after ester hydrolysis, attaching another fragment **27** through a second amidation. To our relief, the final macrocyclization under Heck cross-coupling condition (Pd(OAc)_2_, NEt_3_, Cs_2_CO_3_) proceeded well to afford the product in 58% yield as a configurationally pure 8*E*-nannocystin. No 8*Z*-isomer could be detected by NMR in the crude reaction mixture. Marked by a Heck-type macrocyclization, this route has since served a valuable conduit for systematic SAR research [15,16,17,18].

## 4. Macrocyclization via Stille Cross-Coupling

The value of Pd-catalyzed cross-coupling in macrocycle formation is further demonstrated in Liu’s total synthesis of nannocystin Ax (**2**) [6], which relies on a Stille-type macrocyclization (Figure 2, method **C**). As is often the case with countless natural product syntheses [19], it is only after certain rounds of trial and error while circumventing unforeseeable impasses that a viable route to **2** was settled eventually [20].

**Scheme 3:**
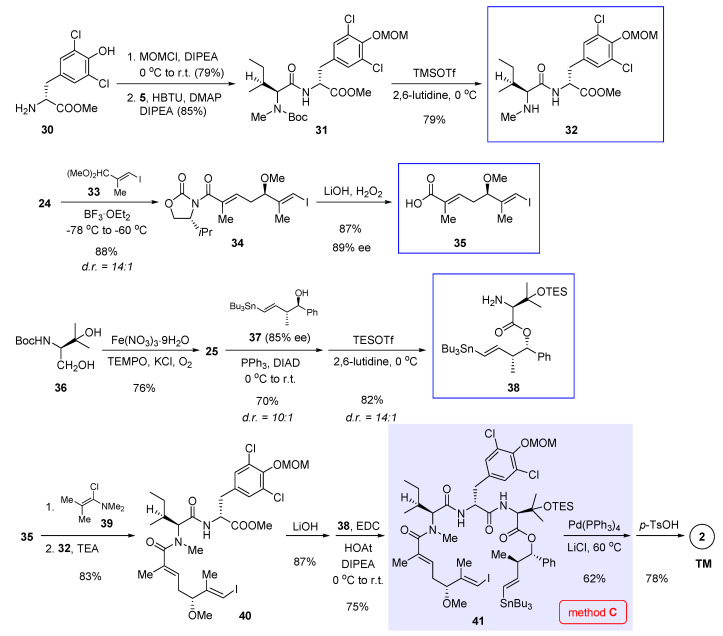
Total synthesis of **2** by Liu et al. featuring macrocyclization via Stille cross-coupling (Figure 2, method **C**) [6].

Liu’s approach makes use of three fragments **32**, **35**, and **38** (boxed in Scheme 3) to assemble the entire molecule. First, MOM protection of chlorinated tyrosine methyl ester **30** and then amide condensation with **5** (structure shown in Scheme 1) gave, after Boc removal, dipeptide **32** as the first building block. Second, vinylogous Mukaiyama aldol reaction, which is instrumental for asymmetric C-C bond formation in the preceding two syntheses (see Section 2 and Section 3) [3,5], again played a key role in the preparation of α,β-unsaturated carboxylic acid **35**; but a somewhat differing aspect of Liu’s synthesis is the use of dimethyl acetal **33**, instead of previously used aldehyde **8** (see Scheme 1), as the electrophilic coupling partner. Thanks to the robustness of Kobayashi’s methodology [11] and subsequent improvement by Hosokawa [14,21,22], the reaction produced **34** in 88% yield with a high level of stereoselectivity (diastereomeric ratio (d.r.) = 14:1). Alkaline hydrolysis aided by hydrogen peroxide cleaved the auxiliary portion to give **35** in 89% ee as the second necessary building block. Preparation of the last building block **38** entailed as the first step an environment-friendly iron-catalyzed oxidation [23] that converted **36** to **25** (structure in Scheme 2) in 76% yield. In the meantime, *trans* homoallylic alcohol **37** was synthesized in 85% ee in accordance with Roush’s asymmetric crotylboration method [24]. The following Mitsunobu esterification built the hindered ester linkage between **25** and **37**. Since the use of protonic acids caused undesirable destannylation, Boc cleavage called for an alternative condition (TESOTf, 2,6-lutidine) to obtain **38** with a diastereomeric ratio of 14:1.

**Scheme 4:**
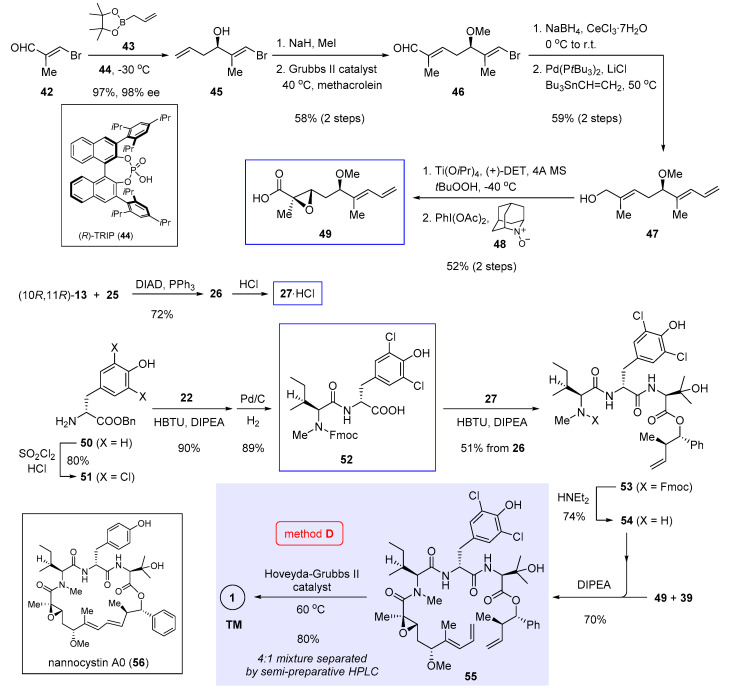
Total synthesis of **1** and nannocystin A0 (**56**) by Wang et al. featuring macrocyclization via ring-closing alkene metathesis (Figure 2, method **D**) [4].

The endgame of Liu’s synthesis commenced with pre-mixing **35** with Ghosez’s reagent **39** [25]**,** from which an acid chloride was generated as a strong acyl donor. Such in situ activated species coupled efficiently with **32** to afford **40** in 83% yield. Earlier, this tactic was employed by Wang et al. during their total synthesis of **1** in order to promote an otherwise refractory amidation reaction (the union of **49** and **54** into **55**, refer to Scheme 4 and the next Section) [4]. Ester hydrolysis of **40**, in conjunction with amidation with **38**, gave open-chain precursor **41**. In the presence of Pd(PPh_3_)_4_ and LiCl at 60 °C, **41** cyclized uneventfully via Stille cross-coupling. At last, global deprotection with *p*-TsOH led to **2** in 78% yield.

## 5. Macrocyclization via Ring-Closing Alkene Metathesis

Ring-closing alkene metathesis as a powerful ring-forming tool was applied by Wang et al. in their total synthesis of **1** [4]. This route (Figure 2, method **D**) seeks piecemeal assemblage of the metathesis precursor **55** from three fragments **49**, **27**, and **52** (boxed in Scheme 4, the structure of **27** is shown in Scheme 2), among which **27** was also employed in our total synthesis [5] as has been covered in Section 3.

Inspired by Antilla’s research [26], homoallylic alcohol **45** was synthesized in 97% yield and 98% ee through enantioselective allylation of (*E*)-3-bromomethacrolein (**42**) with pinacol allylboronate **43** catalyzed by axially chiral phosphoric acid (*R*)-TRIP (**44**). Methylation and alkene cross-metathesis with methacrolein extended the terminal alkene of **45** into an α,β-unsaturated aldehyde. Regioselective Luche reduction of the resulting **46** followed by Stille cross-coupling with Bu_3_SnCH=CH_2_ gave rise to allylic alcohol **47**, onto which Sharpless asymmetric epoxidation installed a stereodefined epoxide. Subsequent oxidation with PhI(OAc)_2_ and AZADO (**48**) afforded the desired fragment **49**. The procedure to synthesize another fragment **27** is almost identical to ours (refer to Scheme 2) and will not be restated.

In the final stage, D-tyrosine benzyl ester **50** was converted to **51** via double chlorination. Succeeding the amidation of **51** with Fmoc protected *N*-methyl-L-leucine **22** (structure shown in Scheme 2), Pd/C-mediated debenzylation yielded dipeptide **52** as the third key fragment. Later, another crucial amide bond was made by coupling **52** to **27**, followed by Fmoc cleavage to generate tripeptide **54**. Union of the two hindered reactants **49** and **54** proved tricky, as a number of peptide coupling reagents failed to effect the desired transformation. Fortunately, by pre-activating **49** with Ghosez’s reagent **39** (structure in Scheme 3), the intermediary participated effectively in condensation with **54** to give **55** in 70% yield. Finally, ring closure of this linear precursor in the presence of Hoveyda-Grubbs II catalyst at 60 °C brought **1** as a mixture of 4:1 stereoisomers. The desired natural 8*E* isomer was isolated with semi-preparative HPLC as the major product in 80% yield. The same route also enabled access to chloro-free nannocystin A0 (**56**, boxed at the bottom left of Scheme 4) by coupling **50** directly to **22** without the initial double chlorination.

## 6. Macrocyclization via Macrolactamization (He’s Approach)

**Scheme 5:**
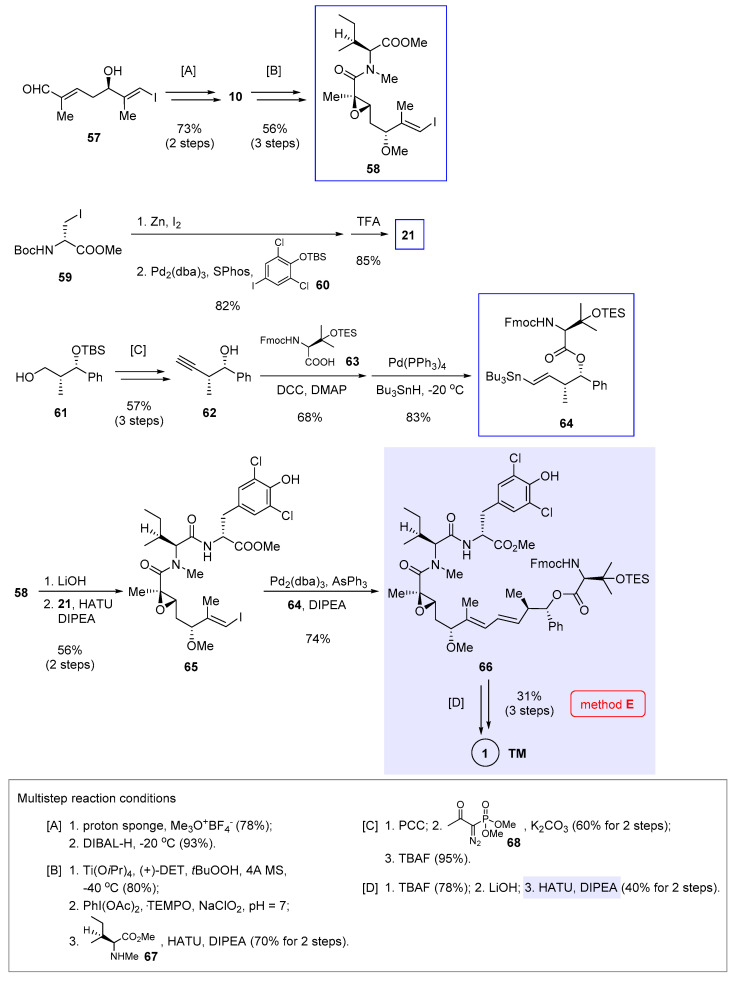
Total synthesis of **1** by Hu and He et al. featuring macrocyclization via macrolactamization (Figure 2, method **E**) [7].

Complementary to transition metal catalyzed reactions, macrolactamization offers a classic solution to generating the nannocystin ring. Still, shifting the cyclization site toward a transannular amide demands a full-length polyketide chain in advance. In 2017, Hu and He et al. communicated their total synthesis of **1 [7]** in which the complete polyketide was prepared from three building blocks **58**, **21** (structure in Scheme 2), and **64** prior to macrolactamization (Scheme 5, method **E**).

Taking advantage of asymmetric vinylogous Mukaiyama aldol reaction developed by Kalesse et al. [27], the authors first prepared chiral alcohol **57** bearing an α,β-unsaturated aldehyde as the starting material. Methylation followed by DIBAL-H reduction (multistep reaction condition [A] at the bottom of Scheme 5) gave allylic alcohol **10** (structure in Scheme 1). Further elaborations include Sharpless asymmetric epoxidation, oxidation of the primary alcohol into a carboxylic acid, and amide coupling with **67** ([B]), thereby producing the first building bock **58**. At the same time, a combination of Pd_2_(dba)_3_ and SPhos ligand enabled Negishi cross-coupling between iodide **59** and an arylzinc species generated in situ from **60**. The tyrosine fragment **21** was then made available via TFA-mediated Boc cleavage. Preparation of the third fragment **64** started with chiral intermediate **61** [28], which underwent a sequence of PCC oxidation, modified Seyferth-Gilbert homologation using **68** [29] and TBS deprotection ([C]) to afford **62**. Key to the nontrivial esterification between **62** and **63** is to mask the latter’s β-hydroxyl with a TES group, otherwise β-lactone would result as a dominant by-product. Similar situation was encountered during Xu and Ye’s synthesis (from **17** to **18** in Scheme 1), wherein the encumbering β-hydroxyl moiety necessitates temporary blocking to ensure azide reduction [3]. After that, Pd-catalyzed hydrostannylation of the terminal alkyne rendered a tributyltin-functionalized (*E*)-alkene in **64**.

To complete the synthesis, **58** was hydrolyzed and coupled with **21**, leading to **65** in 56% yield with simultaneous loss of the phenolic TBS group. After testing various catalysts for the intermolecular Stille cross-coupling between **64** and **65**, an optimal ensemble of Pd_2_(dba)_3_, AsPh_3_ [30], and DIPEA was identified to give the product **66** in 74% yield. Removing both the TES and Fmoc groups, followed by ester hydrolysis, produced a linear intermediate. The ensuing macrolactamization delivered the target molecule **1** in 40% yield over two steps ([D]).

## 7. Macrocyclization via Macrolactamization (Kalesse’s Approach)

**Scheme 6:**
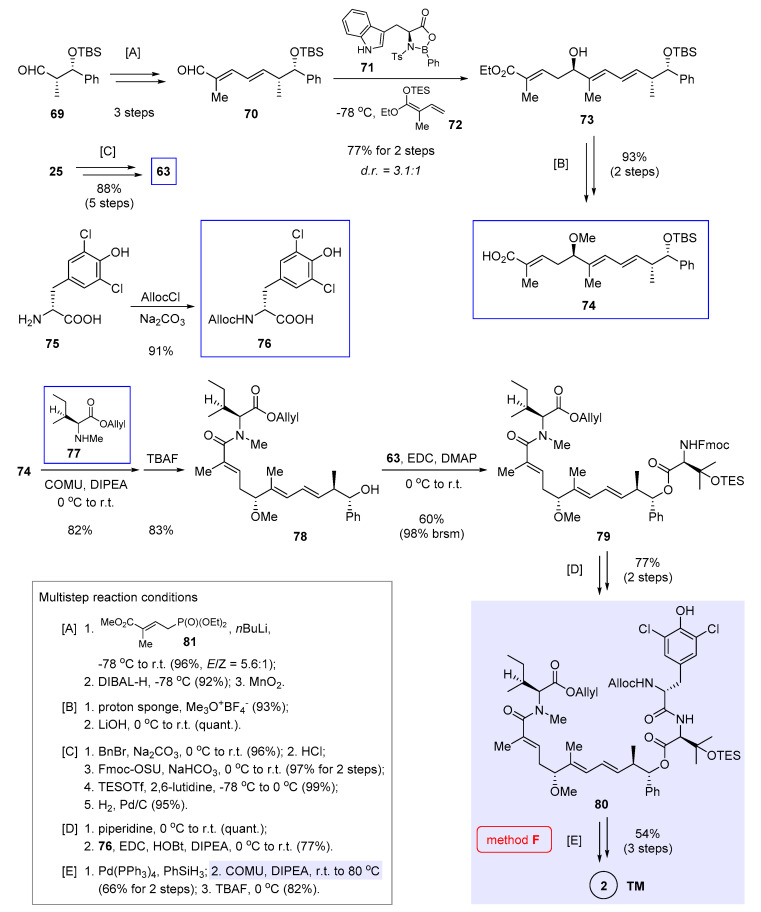
Total synthesis of **2** by Kalesse et al. featuring macrocyclization via macrolactamization (Figure 2, method **F**) [8].

Concurrent to Hu and He’s work [7] shown in the previous section, Kalesse et al. demonstrated the viability of macrolactamization in nannocystin synthesis by utilizing a different scissile peptide bond (Figure 2, method **F**) [8]. Distinctive of their approach is a joint usage of asymmetric vinylogous Mukaiyama aldol condensation [13,31,32] and vinylogous Horner-Wadsworth-Emmons (HWE) reaction [33] in order to access the polyketide segment **74**, to which three amino acids **63**, **76**, and **77 [34]** (boxed in Scheme 6) are added in a stepwise fashion. The synthesis is concluded with macrolactamization and deprotection.

With this in mind, assembly of the polyketide portion was executed first. Thus vinylogous HWE reaction of **69 [28]** with deprotonated phosphonate **81** fared well with good stereoselectivity (*E*/*Z* = 5.6:1). The elongated aldehyde **70** was obtained via DIBAL-H reduction and oxidation with MnO_2_ (multistep reaction condition [A] at the bottom left of Scheme 6). Under the influence of chiral Lewis acid **71** developed in-house [31], vinylogous Mukaiyama aldol reaction of **70** and **72** at −78 °C afforded **73** in a good yield (77% over 2 steps) but with moderate diastereoselectivity (d.r. = 3.1:1). Since the two diastereomers could not be easily separated, purification was deferred to later steps. Even so, the advantage of applying chiral oxazaborolidinones such as **71** to this kind of transformation can still be appreciated. As noted independently by Liu et al. [20], an alternative condition (TiCl_4_, −78 °C) [11] failed to enforce coupling between **70** and **24** (structure in Scheme 2), in spite of its applicability in two earlier nannocystin syntheses (from **7** to **9** in Scheme 1, from **24** to **10** in Scheme 2) [3,5]. To continue, methylation and ester hydrolysis ([B]) generated **74** as an unstable intermediate, which was immediately used in the next step to avoid decomposition on storage. In parallel, protected amino acids **63** (structure in Scheme 5) and **76** were prepared from **25** (structure in Scheme 2) and **75** respectively

Ideally, optimal efficiency will be realized if a full-length tripeptide could add to the polyketide fragment **74**, rather than introducing three discrete amino acids one at a time. Yet in reality this idea proved unworkable, even in the case of a shorter dipeptide. Echoing this result, Liu et al. were unable to modify the N- or C-terminus of their synthetic tripeptide intermediates when exploring a feasible route to **2** [20]. It was suspected that the tripeptide might assume a conformation unfavorable to amide condensation. Accordingly, these three amino acids (**77**, **63**, **76**) had to be attached to the polyketide chain one by one. 

In detail, the first condensation occurred between **74** and **77**, for which a number of peptide coupling reagents were evaluated and only COMU gave a satisfactory yield of 82%. After removing the TBS group with TBAF, the modified polyketide **78** was linked to a second amino acid **63** via esterification. The product **79** was isolated in a moderate yield (60%), albeit most of unreacted **78** could be recovered. Fmoc deprotection and then amidation with **76** led to the masked linear precursor **80** in 77% yield ([D]). This step was found capricious, because upon scale-up the yield dropped considerably with concurring erosion of stereochemical integrity. Future endeavor to optimize these two amidation reactions shall improve the robustness of this route. After Pd-mediated reductive cleavage of the Allyl and Alloc groups of **80**, the pivotal macrolactamization proceeded smoothly to give, after TES deprotection, nannocystin Ax (**2**) in 54% yield over 3 steps ([E]).

## 8. Macrocyclization via Ring-Closing Alkyne Metathesis

**Figure 3:**
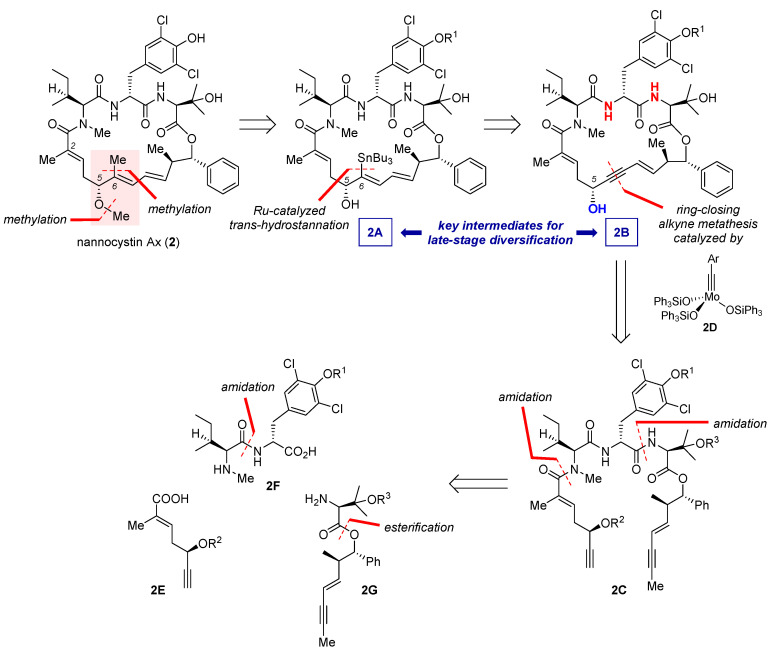
Retrosynthetic analysis of **2** featuring a motif-oriented late-stage diversification strategy.

Total synthesis of bioactive natural products is a prelude to SAR study [35], which helps to determine essential pharmacophores and structural redundancy so that the lead structure could evolve toward better drug-like properties. As the research objective transits from attesting synthetic accessibility to figuring out an SAR trend regarding a specific subdomain, it is sometimes imperative to design an innovative route to meet the diversity-oriented need. This point is elegantly illustrated by Fürstner’s motif-oriented total synthesis of **2** and related non-natural analogues [9]. Unlike the earlier syntheses [3,4,5,6,7,8] aiming at quick access to the nannocystin framework (see Section 2, Section 3, Section 4, Section 5, Section 6 and Section 7), this study takes inspiration from Hoepfner’s docking study [2] suggesting that held by a hydrophobic pocket on the eEF1A surface, the methyl substituent of 6*E*-alkene from the nannocystin polyketide segment might play a key role in target engagement. On the other hand, negligible impact on drug-target binding would be conceived of the adjacent 5*R*-methoxy ether, since it supposedly projects away from the protein surface. Along this thought, structural permutation of **2**, a close congener of **1** with comparable potency, at its 6*E*-alkene as well as the flanking 5*R*-methoxy ether (highlighted in Figure 3) would permit fine-tuning of on-target binding affinity and physicochemical properties.

### 8.1. Retrosynthetic Analysis

Retrosynthetically, **2A** was decided as an advanced intermediate (Figure 3), whose C6 tributyltin unit could diversify into multiple substituents such as a methyl in **2**. In turn, **2A** would be available via Ru-catalyzed *trans*-hydrostannation of the corresponding propargylic alcohol **2B**. Due to Fürstner’s seminal works pioneering the use of [Cp*Ru]-based catalysts (Cp* = η^5^-C_5_Me_5_) [36,37,38,39,40,41], reactions of this sort now proceed with well-defined stereo- and regioselectivity, and as such have found important applications in total synthesis [42]. In the context of nannocystin synthesis, however, the authors cautioned a caveat of applying this strategy. Specifically, there resides an uncertainty of whether and/or to what extent the two weakly acidic transannular amides (red in **2B**, Figure 3), due to their through-space proximity, will compromise the catalyst-steering capacity of the C5 propargylic alcohol (blue in **2B**, Figure 3). Earlier studies present ample evidence that apart from hydroxyl groups, other protic functionalities such as amides and sulfonamides are likewise competent to sway this catalytic process [38,39]. Hence it is worthwhile to find out the actual magnitude of influence by these proximate amides on Ru-catalyzed *trans-*hydrostannation. 

Moving backward, the enyne-bearing macrocycle of **2B** could be built via ring-closing alkyne metathesis (RCAM) [43,44] of its acyclic counterpart **2C** using silyloxy-based molybdenum alkylidyne complex **2D** as the catalyst (method **G**, Figure 2). Discovered by Fürstner et al. in 2009, **2D** and its benchtop stable precursor represent a latest generation of alkyne metathesis catalysts with outstanding reactivity, functional group compatibility, and operational practicality [45,46,47,48]. Since the advent of these broadly applicable catalysts, RCAM in alliance with postmetathetic elaborations has become a privileged methodology [49,50] in the total synthesis of stereodefined polyunsaturated macrocycles [51,52,53,54,55,56,57,58]. As for the present study, **2A** and **2B** constitute two versatile intermediates for diversification. Subsequent disconnection is straightforward in that the open-chain precursor **2C** could stem from three distinct fragments **2E**, **2F**, and **2G**, among which the latter two would be assembled respectively from amidation and esterification of more basic building blocks.

### 8.2. Forward Synthesis

**Scheme 7:**
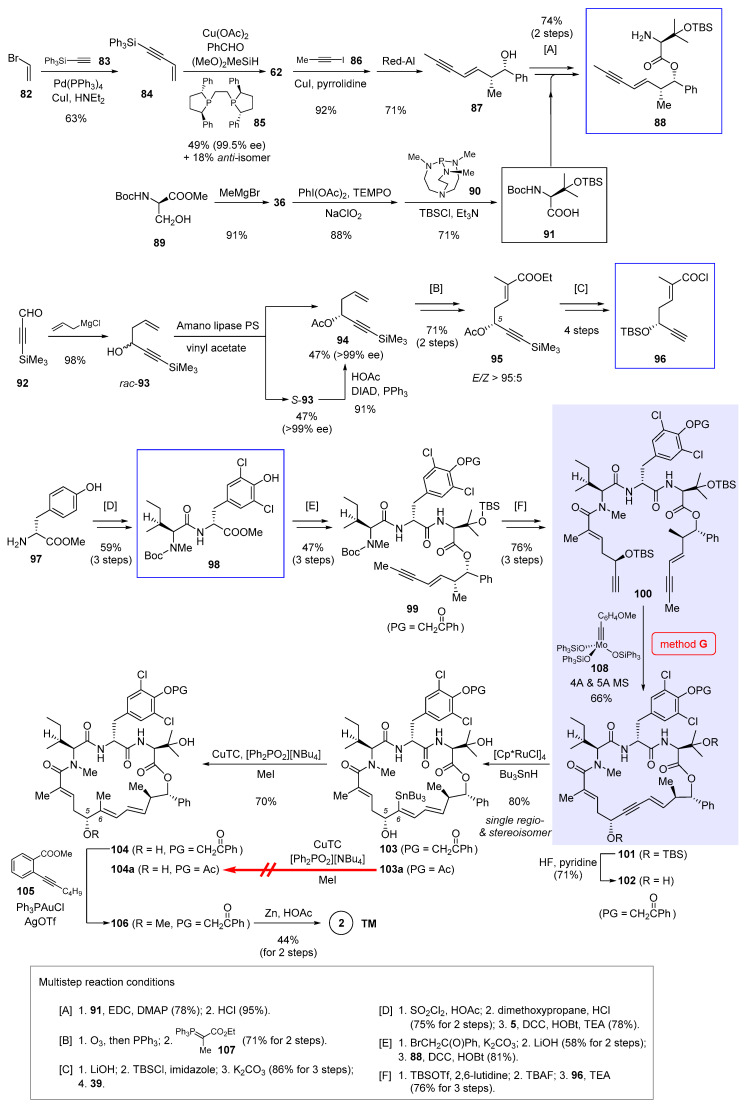
Total synthesis of **2** by Fürstner et al. featuring macrocyclization via ring-closing alkyne metathesis (Figure 2, method **G**) [9].

The first stage of the synthesis [9] as shown in Scheme 7 involves preparation of three key fragments **88**, **96**, and **98** (boxed in Scheme 7). To begin, Sonogashira cross-coupling merged vinyl bromide **82** and silyated alkyne **83** into enyne **84** in 63% yield. Borrowing a new methodology developed by Buchwald et al. [59], **84** underwent Cu-catalyzed asymmetric carbonyl addition to benzaldehyde, in which synergistic actions of a copper-hydride species and its coordinating chiral ligand (*R*,*R*)-Ph-BPE (**85**) imparted excellent optical purity (99.5% ee) to the adduct **62** (structure shown in Scheme 5). Notwithstanding a modest yield (49%) and diastereoselectivity (d.r. = 2.8:1), this two-step sequence compares favorably with an alternative multistep synthesis depicted partially in Scheme 5 (from **61** to **62**). Then a second Sonogashira cross-coupling of **62** with iodopropyne **86**, followed by hydroxyl-directed semireduction with Red-Al, gave rise to enyne **87** in 65% yield over 2 steps. Simultaneously, doubly protected β-hydroxyvaline **91** was supplied in 3 steps from serine derivative **89**. Union of **87** and **91** occurred via esterification, with subsequent Boc removal to give **88** as the first key building block (multistep reaction condition [A] at the bottom of Scheme 7). 

Construction of the second building block **96** started with non-stereoselective allylation of aldehyde **92**. Since attempts at asymmetric allylation failed to bring about satisfactory results, enzymatic resolution was invoked to kinetically separate *rac*-**93** into enantiomerically pure **94** and *S*-**93**. While this single-step transformation produced **94** in no more than 50% yield, the unwanted *S*-**93** could be recycled to **94** via a stereospecific Mitsunobu reaction. In combination, the total yield of this enzymatic resolution procedure reached a satisfactory level of 90%. Next, ozonolysis of the terminal alkene of **94** into an aldehyde, followed by Wittig reaction with phosphorus ylide **106** ([B]), led to **95** with excellent *E* selectivity (*E/Z* > 95:5). Further elaborations ([C]), including (1) ester saponification that concomitantly deacylated the C5 alcohol, (2) TBS reprotection of the exposed chiral alcohol, (3) hydrolytic cleavage of the capping TMS group, and (4) activation of the carboxylic acid with Ghosez’s reagent **39** (structure in Scheme 3), gave acid chloride **96** primed for the following amidation. Preparation of dipeptide **98**, the third building block, was at the same time conducted from tyrosine ester **97** in three steps ([D]), during which Boc-protected *N*-methyl isoleucine **5** (structure in Scheme 1) was included. 

With all three necessary fragments in hand, it is time to move on to the next stage, namely, to integrate them into a proper cyclization precursor. This was set out by masking the tyrosine phenol of **98** with a phenacyl group (PG = CH_2_C(O)Ph). Though counterintuitive at first glance, the choice of this unorthodox moiety turned out crucial in downstream elaborations. If an acetyl group was used instead, the synthesis could advance without complication until the very step of Cu-mediated C6-methylation (from **103a** to **104a**, Scheme 7), where it was prone to transannular rearrangement with consequent elimination of the acetoxyl moiety to render an allene by-product.

Continuing the synthesis, ester hydrolysis of the phenacyl-protected dipeptide and amidation with **88** created tripeptide derivative **99** ([E]). To bring in the missing piece **96**, **99** was treated with large excess of TBSOTf and 2,6-lutidine so that Boc deprotection occurred with simultaneous *N*-silylation. Aqueous work-up with TBAF liberated the tripeptide N-terminus, which coupled efficiently to acid chloride **96** and gave the linear compound **100** ([F]). In the presence of molybdenum alkylidyne catalyst **108** (corresponding to **2D** in Figure 3), RCAM succeeded in forging the 21-membered ring in 66% yield. This result once again exemplifies the robustness of silanolate-ligated molybdenum catalysts in building heavily functionalized macrocycles. Removal of the TBS group then afforded **102** carrying the requisite propargylic alcohol for directed hydrostannation. On the premise of slowly introducing Bu_3_SnH into the reaction mixture, the Ru-catalyzed addition proceeded cleanly to deliver **103** as the only detectable regio- and stereoisomer in 80% yield. 

**Figure 4:**
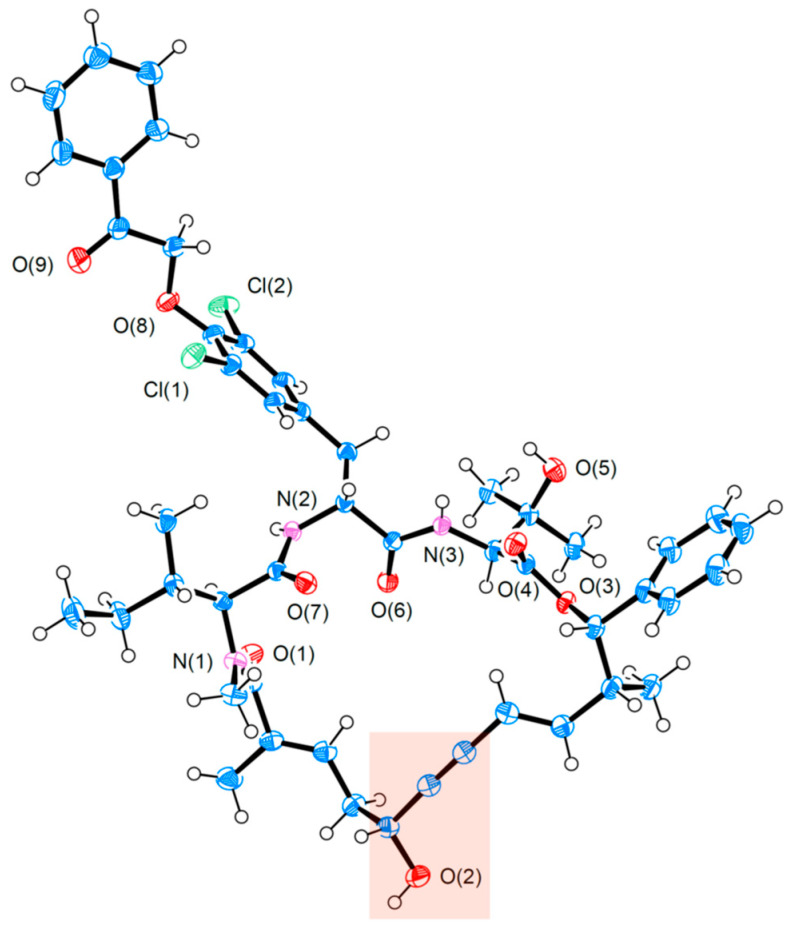
X-ray single crystal structure of the key intermediate **102** during Fürstner’s total synthesis of **2** [9], with its propargylic alcohol highlighted. (ORTEP representation is drawn at 50% probability for thermal ellipsoids.).

On account of such extraordinary selectivity irrespective of two potentially disruptive amides, the authors determined the X-ray single crystal structure of **102** (Figure 4). Obviously, the propargylic alcohol subunit (highlighted red) is situated within a relatively open hemisphere of the macrocycle vis-à-vis the northern side comprising a densely substituted tripeptide. This unbalanced steric environment has dual consequences: on the one hand, it grants access to the bulky [RuCp*]-based catalyst, allowing it to approach the enyne substructure from below so as to be precisely guided by the C5 alcohol for desirable selectivity; on the other, buried inside the congesting tripeptide hemisphere, the two protic amides become less likely to extend their influence on the Ru catalyst. Overall, the solid-state structure of **102** provides a concrete rationale for the observed reaction outcome. Of further note is the efficacy of sequential RCAM and directed *trans*-hydrostannation in constructing macrocycles with a stereodefined *E*-alkene. While RCM of dienes has been widely applied to preparing various-sized rings, a remaining challenge is to control the *E/Z-*selectivity of the nascent alkene, not to mention its indiscriminating reactivity toward alkenes and alkynes [43].

**Figure 5:**
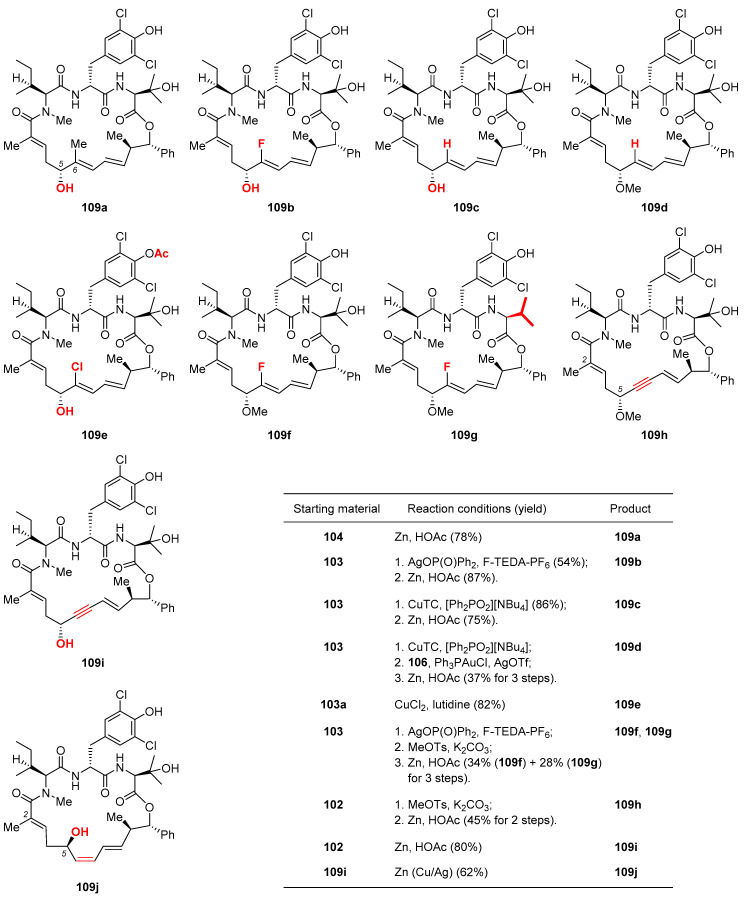
Novel nannocystins **109a**–**109j** prepared during Fürstner’s motif-oriented total synthesis of **2** [9]. The inset table catalogs their preparation conditions.

Toward completion of the synthesis, **103** was subjected to Cu-promoted C6-methylation [60], which produced trisubstituted alkene **104** with intact *E* geometry. Surprisingly, the penultimate step of methylating the C5 alcohol appeared quite challenging, as several commonly used conditions (e.g., MeI/Ag_2_O, [Me_3_O]BF_4_, MeOTs/K_2_CO_3_) failed to produce **106** with decent reliability. Considerable experimentation led to the discovery that base-free gold-catalyzed cyclization [61] of **105** released a methyl cation, which was in no time trapped by the C5 alcohol of **104** to give the methyl ether **105**. In the end, reductive cleavage of the phenacyl group with Zn dust afforded **2** in 44% yield over 2 steps. From the late-stage intermediates **102** and **103**, further diversification provided an array of novel nannocystins **109a**–**109j** (Figure 5) for a focused SAR study.

## 9. Conclusions

All roads lead to Rome. This old adage is true of natural product total synthesis. With a fixed target in mind and an ever expanding toolkit of useful reactions at hand, there can be more than one route to conquer it. Nevertheless, all such roads are different, which is likewise true of total synthesis, as demonstrated by the seven nannocystin total syntheses. Though each approach is uniquely creative, certain approaches turn out more convenient for structural variation. Based on a robust Heck macrocyclization [5], we have been able to conduct systematic SAR study [15,16,17]. Meanwhile, having succeeded in a macrolactamization strategy [7], He et al. later opted for our Heck macrocyclization approach in a subsequent SAR study [18]. This transition reflects the value of synthetic practicality in diversity-driven synthesis. Yet with this strategy, it is still cumbersome to modify the polyketide C5-C9 segment, which according to docking calculation is situated at the forefront of target engagement. Fortunately, Fürstner et al. came up with the motif oriented strategy to tackle this challenge. RCAM in coalition with Ru-catalyzed *trans*-hydrostannation keyed facile access to a library of novel analogues, thereby enriching our knowledge on the SAR of nannocystins. In this case, complementarity in synthetic strategies translates smoothly into that for structural diversification. Both methods, the target-oriented and the motif-oriented, are therefore indispensable for gaining a comprehensive SAR trend, the very road map guiding natural product based drug discovery.
